# Development of a Bicistronic Vector for the Expression of a CRISPR/Cas9-mCherry System in Fish Cell Lines

**DOI:** 10.3390/cells8010075

**Published:** 2019-01-21

**Authors:** Sebastian Escobar-Aguirre, Duxan Arancibia, Amanda Escorza, Cristián Bravo, María Estela Andrés, Pedro Zamorano, Víctor Martínez

**Affiliations:** 1 FAVET-INBIOGEN, Facultad de Ciencias Veterinarias y Pecuarias, Universidad de Chile, Avda. Santa Rosa, 11735 Santiago, Chile; s.escobar.a@veterinaria.uchile.cl (S.E.-A.); cl.bravo.v@gmail.com (C.B.); 2 Department of Cellular and Molecular Biology, Faculty of Biological Sciences, Pontificia Universidad Católica de Chile, 7520245 Santiago, Chile; daarancibia@uc.cl (D.A.); apescorza@uc.cl (A.E.); mandres@bio.puc.cl (M.E.A.); 3 Departamento Biomédico, Facultad de Ciencias de la Salud; Instituto Antofagasta, Universidad de Antofagasta, Avenida Angamos 601, 1240000 Antofagasta, Chile; zamorano@gmail.com

**Keywords:** CRISPR/Cas9, U6 promoter, fish cells

## Abstract

The clustered regularly interspaced short palindromic repeats/CRISPR-associated protein 9 (CRISPR/Cas9) system has been widely used in animals as an efficient genome editing tool. In fish cells, the technique has been difficult to implement due to the lack of proper vectors that use active promoters to drive the expression of both small guide RNA (sgRNA) and the *S. pyogenes* Cas9 (spCas9) protein within a single expression platform. Until now, fish cells have been modified using co-transfection of the mRNA of both the sgRNA and the spCas9. In the present study, we describe the optimization of a new vector for the expression of a CRISPR/Cas9 system, designed to edit the genome of fish cell lines, that combines a gene reporter (mCherry), sgRNA, and spCas9 in a single vector, facilitating the study of the efficiency of piscine and non-piscine promoters. A cassette containing the zebrafish U6 RNA III polymerase (U6ZF) promoter was used for the expression of the sgRNA. The new plasmid displayed the expression of spCas9, mCherry, and sgRNA in CHSE/F fish cells. The results demonstrate the functionality of the mammalian promoter and the U6ZF promoter in fish cell lines. This is the first approach aimed at developing a unified genome editing system in fish cells using bicistronic vectors, thus creating a powerful biotechnological platform to study gene function.

## 1. Introduction

Recently, a new gene-editing system with high targeting efficiency and low cell toxicity, known as clustered regularly interspaced short palindromic repeats (CRISPR)/CRISPR-associated protein 9 (Cas9), has been widely applied across species [1]. CRISPR is based on the prokaryotic endonuclease Cas9 which, when combined with a specific small guide RNA (sgRNA), generates a double-strand break (DSB) in the DNA of invading genomes. This system, used by bacteria against invading bacteriophages and conjugating plasmids, has proved to be a powerful and precise tool editing specific regions of the eukaryotic genome [2]. Here, DSBs are repaired by homology-directed repair (HDR) or by the non-homologous end-joining (NHEJ) pathway, which ultimately leads to insertion/deletion (indel) mutations thereby allowing the edition, insertion, or deletion of genes in eukaryotic genomes [3,4]. A major advantage of CRISPR over zinc-finger nucleases (ZFNs) and transcription activator-like effector nucleases (TALENs) is that the only component that needs to be specifically designed is the sgRNA, which considerably decreases the cost and simplicity of the entire process [4] in a wide range of organisms, generating knockout models fast and efficiently [3,4,5]. The CRISPR/Cas9 system has been used in fish to generate lines of site-directed mutations [6,7,8,9,10]. Editing is usually accomplished by the joint delivery of the Cas9 system and the sgRNA to a single cell fish embryo, and has been successfully carried out in laboratory models such as zebrafish [6] and medaka [11], and in commercial fish species such as Atlantic salmon [7,10] and tilapia [8,9]. In contrast, there are few reports describing the editing of fish cell lines by the CRISPR/cas9 system because co-expression and joint delivery are challenging due to low transfection efficiency [12]. One approach to gene editing in fish cell lines was achieved by stably expressing Cas9 in the modified CHS/F fish cell line and then independently introducing sgRNAs by lipofection, but this resulted in a low transfection rate [12]. Similarly, in medaka cell lines genome editing using the CRISPR/Cas9 system by ribonucleoprotein (RNP) complex was carried out using electroporation [13]. However, this methodology has been difficult to implement in practice due to the lack of fish-specific promoters within a single vector that can drive the co-expression of sgRNAs and the Cas9 protein, and limitations of high throughput delivery into individual cells. Therefore, this study addresses these limitations by presenting the first analysis encoding the protein *S. pyogenes* Cas9 (spCas9) driven by short EF1alpha (EFS-NF) promoter in a bicistronic cassette using mCherry as a reporter gene, in which the self-cleavage mechanism of 2A peptide sequence was functionally recognized in fish cell lines. To achieve the expression of the sgRNA, a cassette containing the zebrafish U6 RNA III polymerase (U6ZF) promoter was cloned. The aim of this study was to develop a powerful gene editing tool that could assist investigations of gene function in fishes, providing information on their role in diseases and other traits, and to improve future biotechnological throughput in aquaculture.

## 2. Materials and Methods

### 2.1. Plasmid Vector Construction

The expression vector LentiCRISPR-Cas9-2A-mCherryU6ZF (LcU6ZF, hereafter) created for fish cell lines was based on the mammalian LentiCRISPR Puro V2 from Feng Zhang´s lab, (addgene plasmid #52961) [14] which was modified in two steps, as follows. To generate LCmCherry V2, the mCherry sequence was obtained from FU-mCherry-w (derived from FUGW) [15] and then digested with *Bsi*WI and *Sac*II restriction enzymes (New England Biolabs, Ipswich, MA, USA). The resulting 0.7 kb amplicon was then purified from the agarose gel (Qiagen DNA extraction kit, Hilden, Germany) and subsequently ligated (T4 ligase, Roche, Basel, Switzerland) into the LentiCRISPR Puro V2 at the site of the discarded puromycin fragment (1.3 kb). Secondly, the full length U6 promoter from zebrafish (U6ZF) was amplified by PCR from genomic DNA *Danio rerio*, using FwU6ZF and RvU6Zf primers. The primers were designed (Table 1) according to Shinya et al. [16], including the *Bsm*BI and *Kpn*I restriction sites, respectively. PCR conditions, using a Pfu DNA polymerase (Invitrogen, Carlsbad, CA, USA), were as follows: 95 °C for 5 min, 40 cycles of 95 °C for 30 s, 56 °C for 30 s, and 72 °C for 0.5 min, with a final extension at 72 °C for 10 min. Finally, the PCR U6 fragment (0.3 kb) was gel-extracted and subsequently cloned into LCmCherry V2 by replacing it with the human U6 promoter region (termed as LcU6ZF). Finally, plasmids were verified by sequencing. The new plasmid sequence generated is included in Supplementary Material 1.

### 2.2. Cloning sgRNA Oligonucleotide in the Novel LcU6ZF Vector 

The insertion of the targeting oligos (EGFP Primers, Table 1) in the LcU6ZF vector was carried out according to the following protocol: first, one microliter (100 μM) of each forward and reverse oligonucleotide (Table 1) was phosphorylated with PNK (New England Biolabs) for 30 min and annealed in annealing buffer (0.4M Tris pH 8, 0.2 M MgCl2, 0.5 M NaCl, 10 mM EDTA pH 8.0) by incubation at 95 °C for 5 min, followed by ramping down to 4 °C /min at 22 °C. Oligonucleotides were diluted (1:200) and ligated into the novel LcU6ZFsgGFP (CGTCTCN▼GCAGAGNNNNN▲) constructed plasmid (plasmid, hereafter). Plasmids were prepared, gel extracted, and isolated using a QIAprep Spin Midiprep Kit (Qiagen, Hilden, Germany). Finally, plasmids were verified by sequencing with sgGFP oligo (Table 1).

### 2.3. Cell Culture and Rates of Transfection

To obtain the transfection rates of the FUGpuro-1D2A-HAW in CHSE/F, 2.5 µg of DNA 6-well plates at high confluency (70–90%) were transfected using Lipofectamine 3000 (Invitrogen, Carlsbad, CA, USA) following the manufacturer’s instructions. Successful transfections were determined by counting the number of GFP positive cells obtained by cell sorting (BD FACSAria II, data not shown) after 96 h using the same parameter described by Dehler et al. [12]. CHSE/F were grown as monolayer at 20 °C in Leibovitz L-15 medium (Invitrogen) supplemented with 10% fetal calf serum (Biological Industries, Kibbutz Beit Haemek, Israel). NOTE: Recently, this cell line has been reassigned as the fish cell line from *Lepomis macrochirus*. Because this finding could still be a matter of controversy, we considered the primary assignation as a Chinook salmon cell line (formerly known as CHSE-214) during the development of this report. 

### 2.4. RNA Isolation and Reverse Transcription (cDNA) of CHSE/F Cells

In order to evaluate the U6 and EFS-NS promoter activity, the total RNA from transfected LcU6ZFsgGFP and non-transfected CHSE/F cells was extracted using TRIzol reagent and treated with RNAse-free DNaseI (Invitrogen, Carlsbad, CA, USA), according to the manufacturer’s instructions. RNA integrity was determined by capillary electrophoresis on the Fragment Analyzer (Advanced Analytical, Santa Clara, CA, USA). The first strand of cDNA was synthesized from 0.5 μg of total RNA using Superscript III Reverse Transcriptase and random hexamers, according to the manufacturer’s instructions (Invitrogen, Carlsbad, CA, USA). The cDNAs were amplified using specific primers (Table 1) on a PCR reaction (GoTaq, Promega, Madison, WI, USA). The PCR products was visualized on agarose gels stained with ethidium bromide.

### 2.5. PCR of CHSE/F Cells Transfected by LcU6ZFsgGFP

Amplifications of sgRNA and transcribed fragment of Cas9 controls (non-transfected) and LcU6ZFsgGFP transfected cells were performed in reactions containing 1 μL of cDNA from an RT reaction with the primers U6ZF_F/U6F_R, Ubq_F/Ubq_R, and FwdGFPPCR/RvgRNAscaffold, respectively, as described in Section 2.1. The PCR primers used for amplifying the targeting sites for each gene are listed in Table 1. The PCR products were fractionated in a 1.5% agarose gel. The ubiquitin gene (*UBQ*) was used as a housekeeping gene, based on a previous work done by Peña et al. [17].

### 2.6. Fluorescent Monolayer of Transfected CHSE/F Cells

After 96 h, cells were washed twice in PBS 1× for 5 min and fixed for 10 min in paraformaldehyde 4% at room temperature. After fixation, the coverslips were permeabilized in PBS/0.05% Triton X-100 and then washed with PBS 1×. Finally, the coverslips were mounted with DAPI (SouthernBiotech). Images were obtained with an Olympus DS-Fi2 epifluorescence microscope operated with the standard QC capture software (Q-Imaging) v4 Nikon. 

### 2.7. Western Blotting

Treated CHSE/F cells were lysed in RIPA buffer containing protease inhibitor cocktail (Sigma-Aldrich, San Luis, Missouri, MO, USA). Proteins were denatured at 95 °C for 5 min, separated by SDS-polyacrylamide gel electrophoresis (10–12%), and then were transferred to nitrocellulose membranes (Millipore, Burlington, MA, USA). The membrane was incubated with PBS 1× containing 5% dehydrated skim milk at 4 °C. The membrane was incubated with anti-mCherry (1:2000, Abexxa, Cambridge, UK), anti-Flag antibody (1:2000, Millipore), anti-H2B antibody (1:2000, Abcam, Cambridge, UK), followed by horseradish peroxidase conjugated goat anti-mouse IgG antibody (1:5000, Invitrogen, Carlsbad, CA, USA). Bands on X-O-mat Blue films (AGFA) were visualized via enhanced chemiluminescence (ECL detection kit; Amersham, Little Chalfont, UK) according to the manufacturer’s instructions. 

### 2.8. Genome Editing on Human Cell Lines Transfected with a Novel LcU6ZF Plasmid

The functional assessment of the LcU6ZFgRNACDNF (cerebral dopamine neurotrophic factor) was carried out in HEK293-T cells due to the high transfection efficiency of this cell line. HEK293-T were cultured in DMEM (Dulbecco’s modified Eagle’s medium; Gibco, Waltham, MA, USA), supplemented with 10% (*v*/*v*) fetal bovine serum (Gibco), 100 units/mL penicillin (Gibco) and 100 μg/mL streptomycin (Gibco), and maintained at 37 °C in an atmosphere of 95% air and 5% CO_2_. Cells were transfected with LcU6ZF mCherry V2 (containing filler fragment as a mock control) and LcU6ZFgRNACDNF with three different sgRNAs (Table 1) using Calfectin agent following the manufacturer’s instruction (Calbiotech, El Cajon, CA, USA). The annealing procedure for three CDNF sgRNAs was in accordance with the protocol described for the GFP oligos above. Forty-eight hours after transfection, the cells were homogenized, and Western blot was performed following the same protocol mentioned above. 

### 2.9. Cell Sorting of CHSE/F Cell Lines Transfected with LcU6ZF β-Actin Plasmid 

There is extensive evidence in the literature on the low transfection rates in these cell lines compared to the classical models such as HEKF-293 T. For this, we cloned new sgRNA and performed experiments in order to study to what extent CHSE/F cells were edited with the construct against the β-Actin gene (accession number: FJ890357, exon 1, see Table 1). This was done according to transfection protocol 2.3. Once the cells were transfected, flow cytometry was used to isolate, sort, and collect an enriched population of mCherry positive cells identified via BD FACSAria II (San Jose, CA, USA). The genomic DNA from transfected and non-transfected CHSE/F cells, respectively, were isolated and purified using a Wizard SV genomics DNA purification system (Promega, Madison, WI, USA). 

### 2.10. High Resolution Melting Analysis of Genome-Edited CHSE/F Cells

In order to confirm gene editing in the CHSE/F cell line, we performed a high-resolution melting (HRM) analysis of DNA for the sorted cells transfected with LcU6β-Actin plasmid expressing mCherry, which is an indication of frameshift mutation or a deletion in the targeted sequence (two replicates each). Primer pairs (β-Actin HRM, Table 1) were designed to amplify the target genome sequence. The PCR reactions were made with 5 μL of the SensiFAST HRM kit 2× (Bioline, Humber Road, London), 0.4 μL of each primer (10 μM) and 2 μL of genomic DNA and water up to 10 μL. The PCR was performed in a Rotor Gene Q (Qiagen, Hilden, Germany). PCR reaction protocol was 95 °C for 3 min, followed by 45 cycles of 95 °C for 15 s and 60 °C for 20 s, and by 95 °C for 60 s, the temperature ramp was increased by 0.1 °C/s from 65 °C to 90 °C, then cooling at 40 °C. Site-directed mutagenesis efficiency was evaluated by analyzing the differences in melting curves of the quantitative PCR products. Curves were analyzed using Rotor Gene Q software (2.3). HRM is more sensitive than standard PCR approaches, enabling early identification of CRISPR-induced indels [18]. 

## 3. Results

### 3.1. Generation of a Novel Bicistronic (Construct) CRISPR/Cas9 System for Modification of Fish Cell Lines

**Figure 1:**
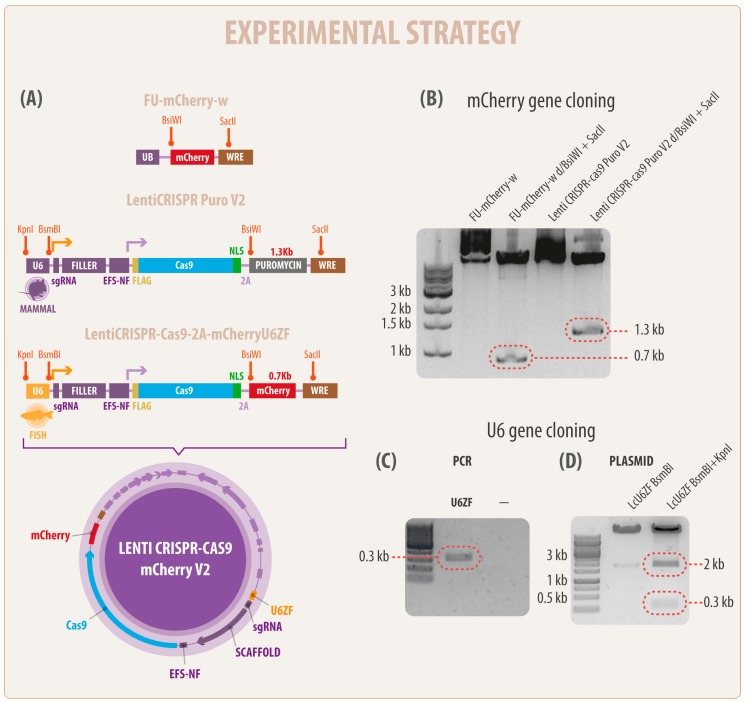
A simplified diagram of the development of a new clustered regularly interspaced short palindromic repeats/CRISPR-associated protein 9 (CRISPR Cas9) vector in fish cells. (**A**) In the left panel and in colors are represented the names and sizes of the regulatory elements including RNA pol III U6 (U6), short EF1alpha (EFS-NS) promoter, small guide RNA (sgRNA), the antibiotic resistance cassette (puromycin), and the mCherry gene. The novel fish U6 promoter (zebrafish U6 RNA III polymerase (U6ZF) ligated in the new vector is represented in yellow. In orange are highlighted the recognition sequences of the restriction enzymes used in this work (*Bsi*WI, *Sac*II, *Kpn*I, and *BsmB*I, respectively). Arrows indicate the downstream activity of U6ZF (yellow) and EFS-NS (violet) promoters. Note that lentiviral elements were omitted in this representation. (**B**) Molecular characterization and isolation of mCherry gene from FU-mCherry-w plasmid (lane 1). In lane 2, a single 0.7 kb fragment (red frame) corresponding to the mCherry sequence was obtained by double digestion (BsiWI and SacII). Lane 3 represents the LentiCRISPR-Cas9 PuroV2 (14 kb) vector, whereas the isolation and removal of puromycin cassette 1.3 kb fragment (red frame) was obtained using the same enzymes mentioned above. (**C**) PCR product of U6 promoter from zebrafish genomic DNA; and (**D**) the new vector LcU6ZF containing the new fish promoter (0.3 kb) highlighted in red, as well as filler fragment (2 kb).

The present study reveals a novel and unified method to express a CRISPR/Cas9 system in the fish cell line CHSE/F. The vector LcU6ZF was adapted from mammalians, to express sgRNAs and the Cas9 nuclease in fish cells. The final plasmid was obtained by replacing, from the plasmid LentiCRISPR Puro V2, the selection marker puromycin and inserting the cDNA of mCherry derived from the plasmid FU-mCherry-w (Figure 1A,B). Additionally, the U6 promoter sequence from zebrafish (data not shown) containing the putative TATA box domain [16] was amplified by PCR (Figure 1C) and cloned in the new LcU6ZF plasmid replacing the U6 mammalian promoter. To assess the correct construction of the LcU6ZF plasmid, a restriction analysis showed the release of the filler fragment of 2 kb (Figure 1D) when digested with the *Bsm*BI restriction enzyme. Additionally, the double digestion with the *Bsm*BI and *Kpn*I restriction enzymes released the U6ZF promoter and the filler fragment indicating that the U6 cassette was properly constructed (Figure 1A–D).

### 3.2. CHSE/F Transfected Cells Expressing both sgRNA and Cas9 

**Figure 2:**
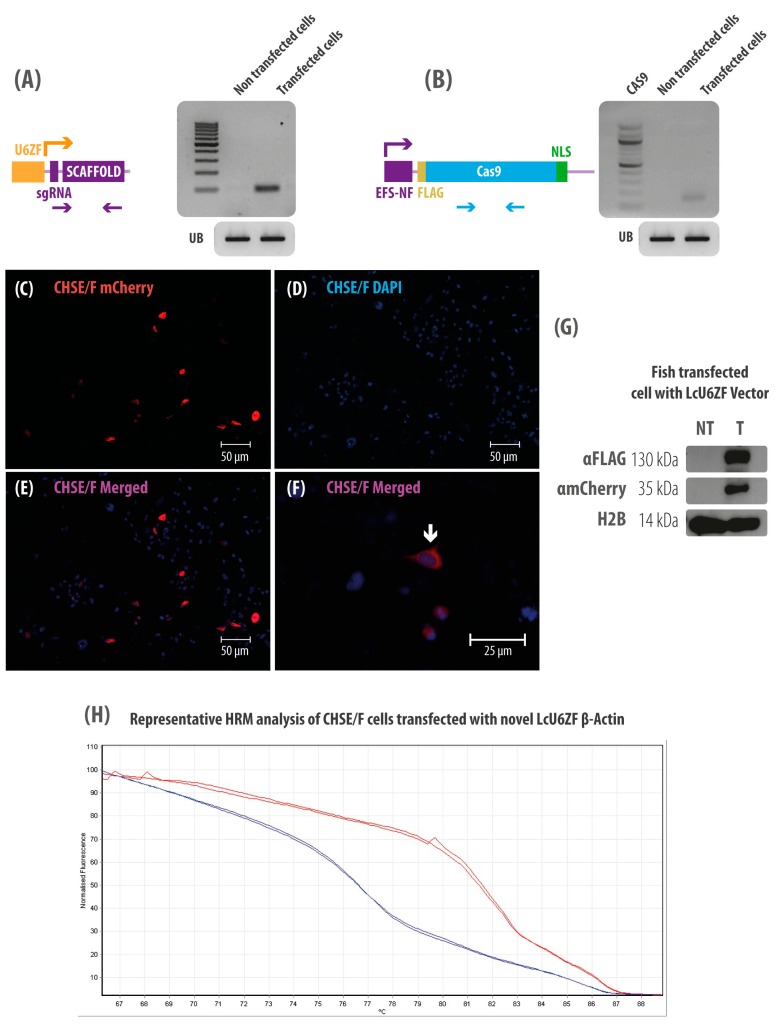
Expression of the sgRNA, Cas9, and mCherry in CHSE/F transfected cells. (**A**,**B**) RT-PCR analysis of the sgRNA (96 bp) and Cas9 (131 bp) expression, respectively. Expression was normalized with the ubiquitin housekeeping gene (image located below, 205 bp). (**C**–**F**) Microscopy of fluorescence imaging. Arrow depicts the mCherry expressing cells at 96 h post-transfection. Bar = 25 and 50 µm. (**G**) Western blot analysis of Cas9 and mCherry in CHSE/F transfected cell. The Cas9 and mCherry proteins were immunodetected using a FLAG antibody (130 kDa) and mCherry antibody (35 kDa), respectively. H2B serves as a loading control (14 kDa). (**H**) Red lines depict the non-transfected melting curve or wild type, whereas the blue melting curve represents the thermal profile of CHSE/F transfected cells. Both samples are biological replicates. Fluorescence difference curves are automatically grouped by the HRM curve analysis system.

The functionality of the U6ZF and EFS-NS promoters to generate the sgRNA and Cas9 mRNA in the CHSE/F cells was assessed by RT-PCR. The results showed that CHSE/F cells transfected with the LcU6ZsgGFP plasmid were able to transcribe (96 bp) sgRNA from the U6 zebra fish promoter (Figure 2A). We also demonstrated that the EFS-NS mammalian promoter, the shorter form of a constitutive elongation factor promoter, was able to drive the expression of Cas9 mRNA (130 bp) in the transfected cells assessed by RT-PCR (Figure 2B), using the ubiquitin gene as a reference gene.

### 3.3. CHSE/F Transfected Cells Express mCherry and Cas9 Protein 

To further evaluate the functionality of the plasmid, an assessment of mCherry and Cas9 protein expression was carried out by fluorescent microscopy and Western blot. CHSE/F transfected cells with the LcU6ZFsgGFP plasmid showed cells conspicuously expressing the mCherry protein, 96 hours after transfection with an efficiency of 10% (Figure 2C–F). These results were consistent with the expression of mCherry by Western blot analysis (Figure 2G). Similarly, the expression of the Flag-tagged Cas9 protein was detected using the αFLAG antibody obtaining a specific signal at the expected size of 130 kDa in the CHSE/F transfected cells. 

### 3.4. Human Cell Knocked-Out CDNF Protein Using the Novel Vector LcU6ZF

Due to the low efficiency of the transfection of the CHSE/F cells, the functionality of the LcU6ZF plasmid was tested in HEK293-T in which we obtained over a 90% transfection efficiency. For this proof of principle, the targeting gene to be modified was chosen to be CDNF, a cerebral dopamine neurotrophic factor that is expressed endogenously in HEK293-T cells. For this purpose, HEK293-T cells were independently transfected with one of three plasmids containing different sgRNAs, designed against the coding sequence of CDNF, in addition to their mock/empty vector (2 kb filler). Forty-eight hours after transfection with LcU6ZFsgCDNF, cells were homogenized and the expression levels of CDNF were analyzed by Western blot (Supplementary Material 2). The knockout for CDNF was effective in one of the three sgRNAs (clone 3) designed, leading to an extremely low expression of protein as observed by Western blot, compared to the mock control. Taken together, it is expected that the plasmid expressing the Cas9 endonuclease, under the EFS promoter, and the sgRNA, under the U6 zebrafish promoter, will be functional in fish cell lines as well. 

### 3.5. Detecting Single-Base Mutations in the Genome of CHSE/F Cells Using LcU6ZF β-Actin Vector by HRM Curve Analysis

The HRM shows a clear difference in the thermal profile between the wild-type and the transfected cells (Figure 2H). The higher peak of temperature for the control group was 82 °C, whereas for the transfected cells, it was at 76.7 °C. The difference of 5.3 degrees Celsius indicates a relatively large change in the targeted gene, a possible indel, which is what we expect in the genome-edited cell line. 

## 4. Discussion

Several strategies based on the CRISPR/Cas9 system have been used to modify genes in different organisms. The current study presents a novel implementation and validation of a CRISPR/Cas9 unified system to conduct high throughput genome editing in fish cell lines (Supplementary Material 1). This system thus permits the investigation of the role of targeted genes in development and diseases. Fish cell lines are a good model to define promoter activity to be used in subsequent studies directed at generating genetic modifications in fish [19,20,21,22]. Therefore, the determination of piscine and non-piscine promoter functions in fish cell lines is crucial for establishing a novel delivery system for CRISPR/Cas9. In this study, we investigated the efficiency of cationic lipofection as a delivery system, using a number of available commercial transfection reagents (DNA carrier), with the aim of improving cell viability (data not shown).

We developed a new optimized unified approach for dual sgRNA and spCas9 expression, given that most CRISPR studies in fish cell lines have been based on the independent use of sgRNA and spCas9 mRNA molecules. 

This approach has the caveat that high concentrations of mRNA could be toxic to the cells, making it difficult to assess the optimized dose for CRISPR expression. In this work, we demonstrated that both U6ZF and EFS-NS promoters were functional in fish cell lines, and plausibly induced indels in CHSE/F fish cells. Furthermore, the bicistronic cassette based on 2A self-cleaving peptide (2A), derived from the porcine teschovirus 1 polyprotein widely use in mammalian cells [23], was able to generate simultaneously the expression of Cas9 and mCherry protein in fish cells as previously predicted [24].

For this study, we used the EFS-NS promoter derived from the core promoter for human elongation factor 1α to direct the expression of the Cas9 protein [14]. The long version of this promoter (EF1) is able to express the green fluorescent protein (GFP) in transgenic rainbow trout very efficiently [21]. However, higher promoter activity can in some cases result in higher gene expression, with an increased toxicity in eukaryotic cells. This situation has been reported for the mammalian cytomegalovirus (CMV) promoter, which has a constitutive activity in CHSE/F fish cells [22]. Therefore, the use of promoters that result in the overexpression of Cas9 nuclease is not recommended, due to the toxicity and off-target effects that may result [25]. In this regard, the system developed here, based on the EFS-NS promoter, is adequate to express the Cas9 protein in the fish cell line CHSE/F. Protein levels of Cas9 were clearly detected by Western blot analysis. Furthermore, no changes in morphology and viability were observed in cells expressing the mCherry used as a reporter of transfection/transduction (data not shown). Such changes are considered major indicators of toxicity [26]. Although the EFS-NS promoter has been previously used in several studies to edit genomes in mammalian organisms [27,28], the activity of this promoter in fish cell lines should be lower than for mammalian cells, due to the evolutionary distance between the taxa. Interestingly, the activity of the EFS-NS promoter was observed directly by mCherry expression (immunofluorescence and Western blot) as the cleavage product induced by the 2A sequence. This proteolytic cleavage site between the two proteins is induced by a 19 amino-acid (aa)-long sequence that mediates the “cleavage” or “translational skip” of polypeptides during translation in mammalian cells [23,24,29]. Indeed, the establishment of a multi-gene expression system (MGES) based on 2A sequences have been widely applied to genetically modified organisms to create new functional or resistant plants [30] and animals [23,24,29]. This is the first report on a fish cell line (the CHSE/F).

We have demonstrated that the U6ZF RNA pol III promoter is functional in other fish species. This result was expected, since U6ZF has been used to induce expression of short hairpin RNAs (shRNAs) for the inhibition of gene expression in other fish studies [16]. The RNA polymerase type III (Pol-III) promoters such as U6 are commonly used to express small RNAs, including shRNAs and single guide RNAs (sgRNAs and scaffold) [16]. Thus, our strategy reveals that the U6ZF promoter could drive the expression of sgRNA against GFP in CHSE/F cells within the framework of a CRISPR/Cas9 system. It must be pointed out that, in addition, we found that this U6ZF promoter is also functional in humans in the assay performed in HEK-293 cells, where we observed the knockout of the CDNF protein (Supplementary Material 2). Thus, our results suggest that the zebrafish U6 promoter could be used in a variety of different cell lines.

Moreover, we have verified the genomic mutations induced by the CRISPR/Cas9 system in CHSE/F cells by HRM analysis. This sensitive approach has been validated in studies aimed at detecting even single base nucleotide polymorphisms [31] and has been successfully used for assessing the success of CRISPR genome editing, in which differences in melting temperature profiles between transfected and non-transfected cells were observed [32].

The CRISPR/Cas9 platform developed in this study is aimed at providing a framework for improving the existing methods for editing fish cell lines. Such editing has proven difficult in practice, given low transfection rates across a number of experiments [12,13]. Since lentiviruses have shown to be a proper platform to deliver the CRISPR/Cas9 in mammalian cells with high efficiency, and given the difficulty of transfecting fish cell lines, the development of a lentiviral [33] for fish cell lines could potentially provide a more efficient throughput screening platform to assess gene function and the role of genes in cell biology and disease. Still, further research is needed in order to assess a proper lentivirus that can effectively be used for improving transfection rates.

## 5. Conclusions

In conclusion, we demonstrated that a new vector that included the zebrafish U6 and mammalian EFS-NF promoters could effectively drive the expression of sgRNA and Cas9 protein, respectively, which in turn was shown to generate mutations in the genomes in vertebrate cell lines. We also demonstrated for the first time the function of a bicistronic expression vector by demonstrating the action of 2A cleavage site in the fish cell lines CHSE/F, indicating the potential role of the developed construct in genome editing using CRISPR/Cas9 in CHSE/F cell culture.

## Figures and Tables

**Table 1:** Oligo and sequences.

Name	Sequence 5′–3′
U6ZF_F [16]	GTGTGGTACCACCTCAACAAAAGCTCCTCGATGT
U6F_R [16]	CAACCGTCTCCGGTGTGGGAGTCTGGAGGACGGCTATATA
GFPA	CACCGGGTGAACCGCATCGAGCTGA
GFPB	AAACTCAGCTCGATGCGGTTCACCC
Ubq_F [17]	GGAAAACCATCACCCTTGAG
Ubq_R [17]	ATAATGCCTCCACGAAGACG
FwdGFPPCR	GGTGAACCGCATCGAGCTGA
RvsgRNAscaffold	ACCGACTCGGTGCCACTTTT
sgRNA1CDNF-A	CACCGACTTGGCGTCGGTGGACCTG
sgRNA1CDNF-B	AAACCAGGTCCACCGACGCCAAGTCC
sgRNA2CDNF-A	CACCTTGTATCTCGAACCCTGTGC
sgRNA2CDNF-B	AAACGCACAGGGTTCGAGATACAAC
sgRNAβactin-A	CACCGCGCCGGAGATGACGCGCCTC
sgRNAβactin-B	AAACGAGGCGCGTCATCTCCGGCGC
βActin HRM-Fwd	GGATCCGGTATGTGCAAAGCC
βActin HRM-Rv	CGTCCCAAAGCCCATCATGAG

## Supplementary Materials

The Supplementary Materials are available online at http://www.mdpi.com/2073-4409/8/1/75/s1.
